# Sex-specific differences in metabolic hormone and adipose tissue dynamics induced by moderate low-carbohydrate and ketogenic diet

**DOI:** 10.1038/s41598-023-43587-9

**Published:** 2023-09-30

**Authors:** Ilya Smolensky, Kilian Zajac-Bakri, Timothy Sasha Odermatt, Catherine Brégère, John F. Cryan, Raphael Guzman, Katharina Timper, Dragos Inta

**Affiliations:** 1 Department of Community Health, University of Fribourg, 1700 Fribourg, Switzerland; 2 Department of Biomedicine, University of Basel, 4056 Basel, Switzerland; 3 Department of Anatomy and Neuroscience, University College Cork, Cork, T12TP07 Ireland; 4 APC Microbiome Ireland, University College Cork, Cork, T12TP07 Ireland; 5 Department of Neurosurgery, University Hospital Basel, 4056 Basel, Switzerland; 6 Department of Endocrinology, Diabetes and Metabolism Clinic, University Hospital Basel, 4056 Basel, Switzerland

**Keywords:** Physiology, Endocrinology

## Abstract

Low-carbohydrates diets are increasingly used to treat obesity and metabolic disorders. A very low-carbohydrate, ketogenic diet is hard to follow and, due to the very high fat content, linked to severe side effects, like hyperlipidemia and atherogenesis. Therefore, a less restrictive, unsaturated fat-based low-carbohydrate diet appears as a promising alternative. Since neither sex differences, nor their effect on specific metabolic hormones and adipose tissue compartments have been investigated thoroughly in these diets, we aimed to analyze their dynamics and metabolic factors in mice. We found a significant sexual dimorphism with decreased body weight and subcutaneous fat only in males on ketogenic diet, while diminished insulin, elevated ghrelin and FGF-21 were present with a differential time course in both sexes. The non-ketogenic moderate low-carbohydrate diet increased body weight and perigonadal fat in females, but induced leptin elevation in males. Both diets enhanced transiently TNFɑ only in males and had no impact on behavior. Altogether, these results reveal complex sex-dependent effect of dietary interventions, indicating unexpectedly females as more prone to unfavorable metabolic effects of low-carbohydrate diets.

## Introduction

Diet-induced obesity (DIO) is a worldwide public health concern, requiring effective and accessible interventions. Low-carb diets are increasingly popular tools for body weight management and treatment of DIO, metabolic and cardiovascular diseases^1,2^. Their composition of and the rate of carbohydrates varies considerably from moderate low-carb (20–40%) diet to very low-carb (5%) ketogenic diet (KD)^2–4^. However, very few studies directly compared the effects of different low-carb diet depending of carbohydrate content.

The KD extends longevity and health span in mice^5^ and is beneficial in various disorders, including obesity^6^. Its efficacy was proven in meta-analyses of clinical trials in both healthy people^7^ and patients with obesity, type 2 diabetes and other metabolic diseases^6^. Initially used as a highly efficient treatment of pharmaco-resistant epilepsy^8^, it shows promising results also in randomized clinical trials of cancer^9^, Alzheimer’s disease^10^, mild cognitive decline^11,12^, chronic pain^13,14^ and Parkinson’s disease^15,16^. However, the common clinical use of KD is largely restricted to children with treatment-resistant epilepsy and strongly hampered in adults, since adherence to KD is a big challenge to many people^17^. Moreover, KD can be accompanied by severe side effects such as growth retardation, nephrolithiasis, hyperlipidemia and atherogenesis^18,19^. Therefore, there are attempts to find a dietary composition which would still have beneficial effects without such a dramatical reduction in carbohydrates and better compliance^1,20^. Moderate low-carbohydrate diets (hereafter mentioned as LCD) with 20–40% of carbohydrates may represent an alternative, but their effectiveness is poorly studied. Both diets effectively reduce blood glucose level, improve glucose tolerance and insulin sensitivity^21^. KD increases serum and liver triglycerides while a moderate LCD improves serum lipid profile and hepatic lipid metabolism potentially being even more beneficial than KD^21^. However, moderate LCDs used in studies resemble often obesogenic high-fat diets (HFD), associated with detrimental effects of high saturated fats on metabolic and mental health^22,23^. Therefore, we used an alternative LCD where saturated fat is replaced with unsaturated fat, known to improve metabolic health^24^. We aimed to analyze the effect of these diets in both sexes, since most rodent studies were done in males while females are excluded due to their resilience to HFD-induced obesity^25^. However, for an effective therapeutic management of obesity in humans the outcome in both sexes needs to be investigated.

## Results

**Figure 1:**
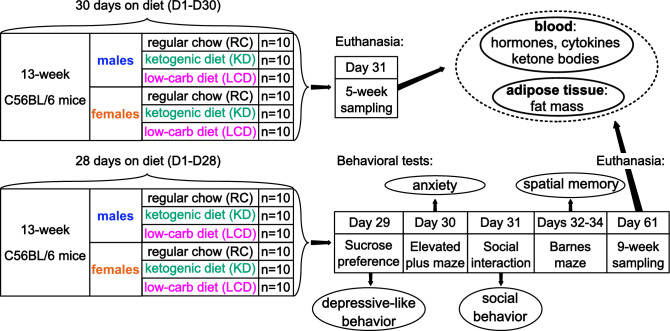
Design of the experiment.

**Figure 2:**
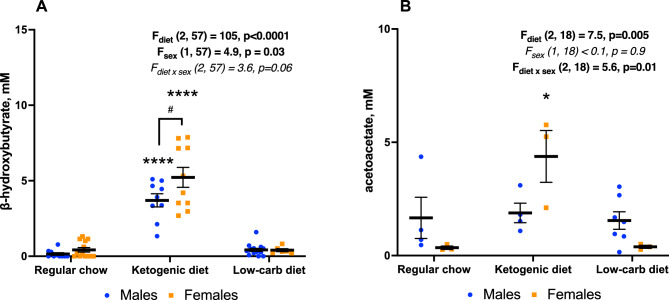
Serum ketone bodies concentration after 1 month on diet. (**A**) β-hydroxybutyrate, (**B**) acetoacetate. Mean ± SEM, two-way ANOVA (diet × sex), *significant difference from RC, p < 0.05, ****p < 0.0001, ^#^significant sex differences, p < 0.05, Tukey test.

**Figure 3:**
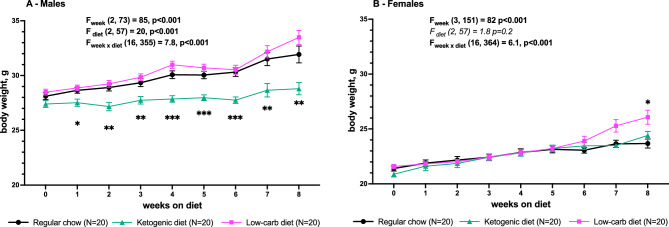
Body weight of male (**A**) and female (**B**) mice during 8 weeks on diet. Mean ± SEM, two-way repeated measures ANOVA (week × diet), *significant difference from RC, p < 0.05, **p < 0.01. ***p < 0.001, Tukey test.

We studied the effect of 1 and 2 months of regular chow (RC), LCD and medium-chain fat-based KD on body weight, white adipose tissue (WAT) distribution, metabolic hormones, cytokines and behavior in adult male and female mice (Fig. 1). We found a significant effect of KD, but not LCD, on blood ketone bodies which was stronger in females—β-hydroxybutyrate (BHB, Fig. 2A) level was higher in KD-fed females than males while acetoacetate (AcAc, Fig. 2B) was elevated only in females (although not all blood samples were analyzed). KD reduced body weight gain over time compare to RC only in males (Fig. 3A) with no effect in females. LCD, on contrary, increased body weight only in females but significant group differences started only after 8 weeks on diet (Fig. 3B).

**Figure 4:**
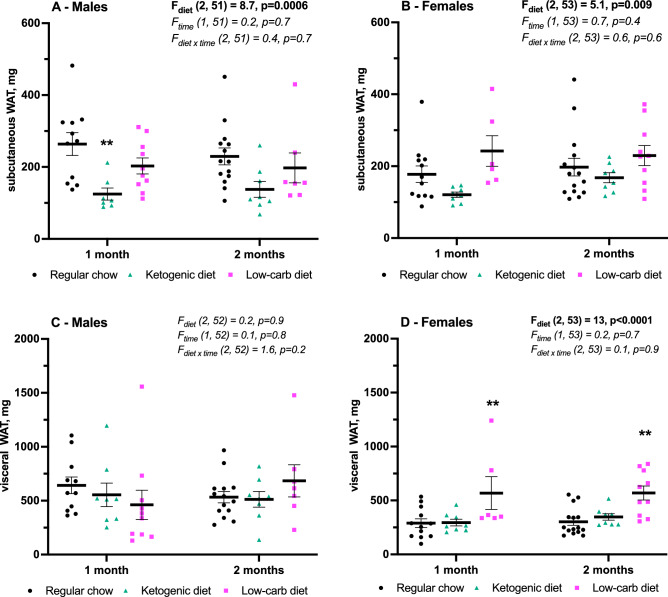
Inguinal (**A**, **B**) and perigonadal (**C**, **D**) fat mass after 1 and 2 months on diet. (**A**, **C**) males, (**B**, **D**) females. Mean ± SEM, two-way ANOVA (time × diet), *significant difference from RC, p < 0.05, **p < 0.01. ***p < 0.001, Tukey test. Analysis of fat samples was not performed in all mice.

The adipose tissue mass (Fig. 4) changed accordingly to the body weight in males and females on KD and LCD. In males KD reduced mass of only subcutaneous inguinal white adipose tissue (iWAT, Fig. 4A) with no effect in females. While significant LCD-induced increase fat mass in females was found only for visceral perigonadal WAT (pgWAT, Fig. 4D). No effect of either diet was found on iWAT in females (Fig. 4B) and on pgWAT in males (Fig. 4C). Note that fat samples not from all mice in each group were analyzed (6 to 15 from 20 in total).

**Figure 5:**
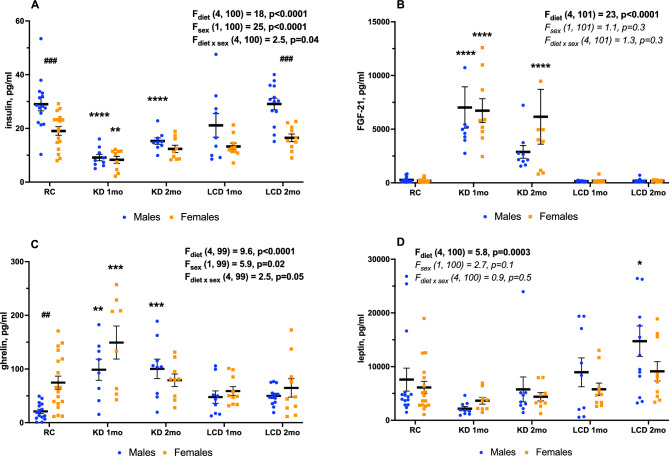
Insulin (**A**), FGF-21 (**B**), total ghrelin (**C**) and leptin (**D**) in serum, Mean ± SEM, two-way ANOVA (diet × sex), *significant difference from RC, p < 0.05, **p < 0.01. ***p < 0.001, ****p < 0.0001, ^##^significant sex differences, p < 0.01, ^###^p < 0.001, Tukey test.

After one month on KD the serum insulin level declined (Fig. 5A) and the FGF-21 level increased (Fig. 5B) in both sexes on KD, but not on LCD. KD-induced changes were present for 2 months for insulin only in males (Fig. 5A), while for FGF-21 only in females (Fig. 3B). Ghrelin was increased in both sexes after one month on KD, but only in males after two months and in none of LCD groups (Fig. 5C), whereas leptin was elevated only in males after 2 months on LCD (Fig. 5D).

**Figure 6:**
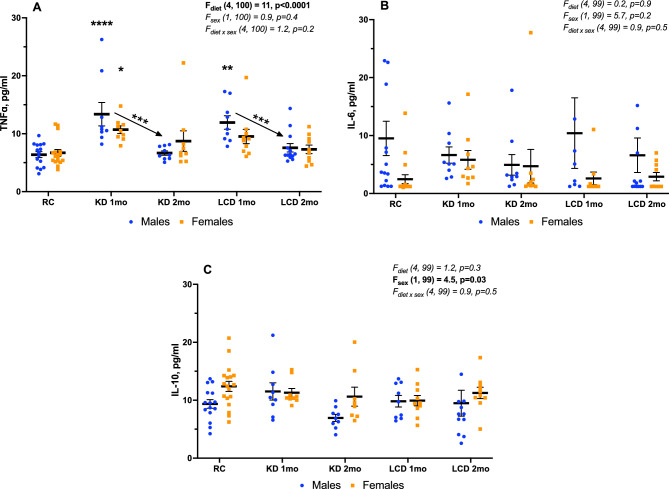
TNFɑ (**A**), IL-6 (**B**), IL-10 (**C**) in serum, Mean ± SEM, Two-way ANOVA (diet × sex), *significant difference from RC or between KD groups, p < 0.05, **p < 0.01. ***p < 0.001, ****p < 0.0001, Tukey test.

The pro-inflammatory cytokine TNFɑ was elevated in serum after 1 month of both KD and LCD but only in males (standard chow 6 ± 1 pg/ml, KD 1 month 13 ± 2 pg/ml, LCD 1 month 12 ± 1 pg/ml, Fig. 6A). After 2 months TNFɑ level decreased significantly compare to 1-month value (KD 2 months 7 ± 1 pg/ml, LCD 2 months 8 ± 1 pg/ml) and returned to basal levels (Fig. 6A). Meanwhile, no changes were found in the levels of the pro-inflammatory IL-6 and anti-inflammatory cytokine IL-10 in none of the treatment groups (Fig. 6B,C).

**Figure 7:**
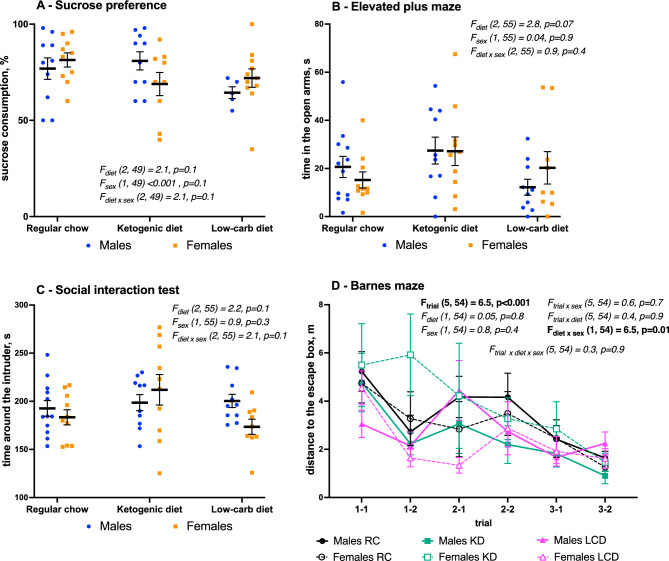
Behavioral effects of diets. (**A**) depressive like behavior in sucrose preference test, (**B**) anxiety in elevated plus maze, (**C**) social behavior in non-reciprocal social interaction test, (**D**) spatial memory in Barnes maze. Mean ± SEM, two-way (**A**–**C**, diet × sex) or three-way repeated measures (**D**, trial × diet × sex) ANOVA.

No behavioral effects were found for KD or LCD on depressive-like behavior in sucrose preference test (Fig. 7A), anxiety in elevated plus maze, (Fig. 7B), social behavior in social interaction test (Fig. 7C) or spatial memory in Barnes maze (Fig. 7D).

## Discussion

Here we report a significant sexual dimorphism of LCD and KD on body weight, WAT compartments, and metabolic hormones, a result which may be important for regarding the use of these diets in humans.

KD-induced ketosis was confirmed by elevated serum BHB, which was higher in females, in line with previous reports^25^. Moreover, AcAc was increased only in females. However, despite more pronounced ketosis, KD had no effect on body weight in females while significantly reduced it gain in males. In addition, whereas females showed no KD-induced changes in fat mass, iWAT was transiently reduced in KD-fed males compare to RC. These results are important given the structural and functional differences between two fat compartments: subcutaneous iWAT acts as a “metabolic sink” receiving and sequestering excess lipid, whereas visceral fat including pgWAT contributes to obesity-associated mortality^26^. Therefore, the lack of effect of KD on pgWAT and the reduction of protective iWAT in males, albeit a body weight effect occurred, question a clear beneficial action of KD. 2-g body weight reduction in males after one month on KD was accompanied by just 0.11 g reduction of visceral pgWAT and 0.14 g reduction in subcutaneous iWAT. In a similar study with 3-week KD in male mice Weber and colleagues found 10-g body weight reduction accompanied by 1 g of fat mass reduction (0.75 g of iWAT, 0.14 g of pgWAT)^27^. Effect of KD on the body weight and fat mass was higher in Weber’s study but proportion of fat mass change to body weight change was similar—10% in their study and 12.5% in ours. However, in Weber’s study iWAT change was fivefold bigger than pgWAT change while equal in our study. Meanwhile, it’s important to note that perigonadal fat by definition is not the same in males in females and therefore might respond differently to the same factors. One study reported that pgWAT removal improved glucose tolerance in both sexes fed with RC while only in high-fat diet-fed obese females^28^. Two studies directly compared KD-induced pgWAT changes between sexes. One month on diet did not induce any changes in either body weight or fat mass in both sexes^29^ while 4-month KD also did not change the body weight in either sex but in males it increased pgWAT mass^30^. Other studies with 4-week KD also reported to increase pgWAT mass and decreased lean mass in females (with unchanged body weight)^31^ and in males (with body weight loss)^32^. However, in our experiments both 1-month and 2-month KD had no effect on pgWAT in either sex. Measures of other fat deposits (retroperitoneal, perirenal, mesenteric) would have made a clearer picture of metabolic effect of KD.

We found that KD boosts FGF-21 production in both sexes, with higher levels and longer persistence in females. FGF-21 is a main metabolic hormone, promoting iWAT expansion, which is determinant for insulin sensitivity^33^. The reason for the discrepancy between iWAT and FGF-21 found here is unclear, suggesting the action of other, yet unknown FGF-21-independent sex-specific factors. The FGF-21 increase was paralleled by an insulin drop, possibly as a direct effect of FGF-21^34^, which was more persistent in males, slightly different from the sex-specific FGF-21 dynamics. KD had no effect of leptin, but increased ghrelin, an effect associated with fasting, reflecting recovery of insulin sensitivity and pgWAT regression in DIO^35^. These results indicate complex sex-specific metabolic actions of KD. Meanwhile, in both sexes KD induced elevation of TNFɑ but no changes in IL-6 and IL-10 level which might reflect some mild pro-inflammatory effect.

One potentially harmful feature of KD might be a low protein contain which can result in protein deficiency-induced side effects. With a fat mass of 75 to 90% in KD, the protein contain is inevitably reduced to 10–15%, however other studies using similar dietary compositions during two^25,36^, three^37^ or four^38^ months have not reported significant protein deficiency-induced detrimental effects. One study comparing two forms of KD with regular (13% of energy) and low (0% of energy) protein contain during 8 weeks in male mice found loss in body weight and lean mass induced by low-protein KD, while body weight gain in regular-protein KD-fed mice^39^. Interestingly, FGF-21 level in plasma was also increased only in the low-protein KD group, while we found it in KD with 10% of protein-derived energy.

LCD unlike KD did not trigger body weight loss, even increasing in females pgWAT and body weight, albeit the latter only after 8-week diet, contrary to the higher DIO susceptibility of males^25^. Few animal studies used diets which were called “low-carbohydrate” with 20%^40^ or 40%^41,42^ of carbohydrates but their authors did not mentioned exact fat composition. These studies reported body weight loss^42^ and improved blood lipid and metabolic profiles^40,41^ suggesting beneficial effect of such moderate low-carbohydrate diets. However, our results suggest that high rate of even unsaturated fats in the diet might increase rather than decrease body weight, at least in females. The reason for this difference is unclear, it may rely in different factors, like the distinct diet composition, in our case with predominantly unsaturated fats in LCD compared to saturated fats in other low carb diets and HFD.

In both sexes, LCD did not induce ketosis and changes in FGF-21, insulin and ghrelin, showing a different metabolic profile than KD and HFD^35,43^. Interestingly, LCD increases leptin, together with TNFɑ (that stimulates its expression), only in males, a result contrasting at a first glance with the WAT dynamics. One possible explanation could be that leptin is produced also independently from the WAT by muscles, where it can increase its own expression^44,45^. This possibility needs to be investigated by future studies. The fact that the iWAT is the main source of leptin^46^ and its reduction by KD reported here, may further explain the difference between the leptin and TNFɑ dynamics in males by LCD and KD, respectively. In contrast, these changes may not occur in females, where no difference in WAT by KD or only a late increase of pgWAT in the course of LCD were found.

One limitation of our study is that due to the high fat content of the experimental diets and the resulting changed food consistency, they were provided in form of paste and not as pellets. Therefore, a precise food consumption monitoring was not possible and the current data have to be interpreted with this practical limitation. Another limitation is that unfortunately, fat samples not from all mice in each group were analyzed (6 to 15 from 20 in total) which might potentially bias the results.

In sum, our data show distinct sex-specific effects of two LCDs on fat tissue distribution, and metabolic parameters. Despite some positive effects, including no behavioural disturbances, our data question their overall benefit, indicating that there is a need for a more differentiated view of beneficial versus deleterious actions based on diet composition. Of note, a recent large prospective clinical study reported that overall LCD and unhealthy (high-saturated fat) LCD were associated with a significantly increased mortality risk, while a healthy (high-unsaturated fat) LCD slightly reduced the risk^47^. Although this study was done in middle age and older probands, which may not correspond to the young adult stage rodent model investigated here, it reveals detrimental effects of LCDs depending on fat composition, raising concerns in comparison with previous evidence^48^. Therefore, future studies are necessary to better understand the underlying mechanisms towards a more rational use of these diets.

## Materials and methods

### Animals

All methods were performed in accordance with the relevant guidelines and regulations. Animal protocol was approved by University of Basel Animal welfare office, Basel-Stadt Cantonal veterinary office and Cantonal ethical commission (license №3094). 13-week-old male and female C56Bl/6J mice (N = 120) (Janvier Labs, Le Genest-Saint-Isle, France) were used in the study. Mice were kept in groups of 4–5 per cage under 12-h light cycle (8 a.m.–8 p.m.) with food and water ad libitum. In order to reduce variation of females outcomes caused by hormonal fluctuations, their estrous cycles were synchronized by Whitten effect (putting male bedding into female cages) twice during the 4 weeks of diet^49^.

### Diets

Three types of experimental chow were used in the study, all of them were produced by Kliba-Nafag (Kaiseraugst, Switzerland). Ketogenic diet (KD) chow had a form of fat paste, low-carbohydrate diet (LCD) had a form of granular quark and regular chow (RC) was a standard pellets crushed into powder to exclude the chewing factor which was reported to affect mental health and neurodevelopment^50,51^. The composition and energy profiles of all three chows are present in the Table 1. Since medium-chain triglycerides (MCT) were reported to have stronger ketogenic effect that long-chain triglycerides (LCT)^52^, we used MCT-based alternative KD instead of LCT-based classic KD. Mice were health checked daily and weighted weekly. Each of six groups (males/females x RC/KD/LCD) included 20 mice—10 were killed by cardiac perfusion after 1 month on diet, 10 underwent behavioral tests and were killed after 2 months on diet.

**Table 1:** The composition of diets.

	Regular chow (RC)	Ketogenic diet (KD)	Low-carb diet (LCD)
Carbohydrates (mass/energy)	59%/55%	3%/3%	20%/17%
Proteins (mass/energy)	19%/32%	10%/7%	35%/23%
Fats (mass/energy)	5%/13%	75%/90%	35%/60%
Saturated fats (% of fat mass)	7%	70%	9%
Monounsaturated fats (% of fat mass)	51%	23%	57%
Polyunsaturated fats (% of fat mass)	32%	7%	36%
Total energy (Kcal/Kg)	3120	7208	5258

During the accommodation week (week 0) experimental chow (SC-powder/KD-paste/LCD-paste) was put into a cage along with standard pellets to reduce stress of switch. After the mouse was put on diet it maintained on it until the end of experiment. Each of six diet × sex groups (n = 10) was further subdivided into two cohorts of different time points after 4 weeks of diet: (1) behavioral tests during days 1–8 and following blood and adipose tissue sampling after 2 months (D60) of diet; (2) blood and tissue sampling after 1 month of diet (D31). Total number of animals used in the experiments was N = 120 (Fig. 1). Mice were weighted three times a week and the average was used as a body weight after each week on diet.

### Behavioral tests

Behavioral studies started after 4 weeks of diet and included 4 tests for anxiety, social and depression-like behaviour and spatial memory.

#### Depression-like behavior: sucrose preference test (SPT)

Each mouse was kept in separate cage with two bottles with water and with 2% sucrose solution. Sucrose consumption was measured during 18 h, from 10 a.m. to 4 p.m.^53^.

#### Anxiety: elevated plus maze (EPM)

A mouse was placed in a center of the maze (35 × 5 cm each arm, 20 cm wall, 50 cm above the floor, 25–30 Lx in the open arms). Time spent in the open arms was measured during 5 min of the test^54^.

#### Social behavior: non-reciprocal social interaction test (SIT)

Only tested mouse was left in its home cage and unfamiliar same-sex intruder mouse was placed under the metal mesh cup to prevent direct contact and potential fights. Time spent near the cup during 5 min of test was calculated as a measure of contact^55^.

#### Spatial memory: Barnes maze (BM)

During 3 days of two 5-min trials (10 min apart) each mouse was placed into a center of brightly illuminated (1000 Lx) 75-cm round arena 80 cm above the floor until it found a way to the escape box under one of 16 round 5-cm holes. The length of walking path from start point to the escape box was used as a measure of spatial learning and memory^56^.

Tests sequence (Fig. 1) was designed to increase stress level. Mice were placed into a maze using a tube to decrease stress, the apparatus was cleaned with ethanol after each mouse, males and females were tested separately. EPM, SIT and BM recordings were analyzed using ANY-Maze software (Stoelting Europe, Dublin, Ireland).

### Sample preparation

At the D35 or D60 mice of corresponding group were anesthetized with ketamine/xylazine. After dissection the skin the inguinal subcutaneous white adipose tissue (iWAT) was collected from left and right sides of the body. Then the abdomen was open to collect perigonadal visceral white adipose tissue (pgWAT) and the rib cage was open to collect blood samples (∼ 0.3 mL) by cardiac puncture. Fat tissue samples were weighted, put into foil and stored at − 80 °C. Blood samples were kept on room temperature for 30 min and then centrifuged at 4 °C and 2000×*g* for 15 min. Serum supernatant was transferred into another tube, kept on dry iced and then stored at − 80 °C.

### Serum biochemical analysis

Ketone bodies (β-hydroxybutyrate, BHB and acetoacetate, AcAc) level was measured in serum samples to estimate ketogenesis after one month (5 weeks) on each of three diets. Ketone Body Assay Kit (Sigma-Aldrich, St. Louis, MO, USA) was used according to the manufacturer instructions. Enzymatic reaction involving β-hydroxybutyrate dehydrogenase requires NADH which absorbance (340 nm) reflects BHB or AcAc concentration. Metabolic hormones (insulin, FGF-21, leptin, total ghrelin) and cytokines (IL-6, IL-10, TNFɑ) concentration in serum samples was measured by U-Plex Metabolic Group 1 (Mouse) Multiplex Assay Kit (Meso Scale Diagnistics, Rockville, MD, USA) according to the manufacturer instructions.

### Statistics

Body weight dynamics was analyzed by two-way RM ANOVA (week x diet) separately for males and females. Behavioral and biochemical results were analyzed by two-way (diet × sex) or three-way (trial × diet × sex for Barnes maze) ANOVA followed by Tukey pair-wise comparisons of corresponding groups in case of significant F-test (p < 0.05). Statistical analysis and graphs were made in GraphPad Prism 9 (GraphPad Software, LLC, Boston, MA, USA). The study is reported in accordance with ARRIVE guidelines.

## Data Availability

Data is available online https://osf.io/s5ync/?view_only=ea61b23db1bd4f108c373ec3cf794a6f.
